# The relationship between arthritis and mild cognitive impairment in different obese metabolic heterogeneity populations-the mediating role of depression

**DOI:** 10.3389/fnut.2026.1653086

**Published:** 2026-02-02

**Authors:** Chen Li, Yitie Chang, Jingxuan Cui, Zhetian Wang, Linru Zeng

**Affiliations:** 1 Jiangnan Hospital Affiliated to Zhejiang Chinese Medical University, Hangzhou, China; 2 The First School of Clinical Medicine, Zhejiang Chinese Medical University, Hangzhou, China

**Keywords:** arthritis, depression, mediation, metabolic heterogeneity, mild cognitive impairment, obesity

## Abstract

**Objective:**

The purpose of this study is to investigate whether depression may mediate the association between arthritis and mild cognitive impairment (MCI) in diverse BMI-based obese populations with multi-parameter metabolic signatures including insulin resistance, inflammatory markers, and lipid metabolism indices.

**Methods:**

The relationships between arthritis, MCI, and depression were investigated in a large-scale study that used logistic regression, subgroup analyses, and mediation analyses. The participants in the study came from obese individuals classified into distinct metabolic obesity phenotypes based on insulin resistance, inflammatory markers, and lipid metabolism.

**Results:**

Arthritis is positively correlated with MCI (OR = 1.86, 95% CI = 1.60–2.17, *p* < 0.001), especially in the metabolically unhealthy overweight/obesity population (OR = 2.27, 95% CI = 1.73–2.97, *p* < 0.001), and depression plays a mediating role in the relationship between arthritis and MCI in this population (proportion of mediation: 17.5%).

**Conclusion:**

The results of this study highlight the potential clinical value of an integrated management strategy combining metabolic regulation, anti-inflammatory treatment, and routine depression screening in obese patients with arthritis to reduce the risk of cognitive decline.

## 1 Introduction

Osteoarthritis (OA) and rheumatoid arthritis (RA) are the two main types of arthritis, a degenerative disease that is closely associated with age. Progression of articular cartilage degradation and loss of subchondral bone structures are shared pathological hallmarks of these disorders. These conditions may impact one or more joints (1). Epidemiological data shows that 31.4% of China’s middle-aged and senior individuals, or around 154.4 million people, suffer from arthritis. Approximately 55% of the world’s arthritis sufferers are 55 and older (2). Population aging has led to a rising prevalence of arthritis, posing a public health challenge. The pain caused by arthritis not only affects patients’ quality of life but also results in reduced work capacity and a substantial increase in both family and societal economic burdens (3, 4). Therefore, identifying factors associated with arthritis and formulating effective prevention and intervention strategies hold significant public health importance.

According to the World Alzheimer’s Report, the incidence of dementia is steadily increasing over the world. In the next 30 years, the number of cases is projected to climb from 46.8 million to 131.5 million (5). Dementia is most often caused by Alzheimer’s disease (AD). Early preventative methods have been shown to be able to avert certain occurrences of AD (6). Mild cognitive impairment (MCI) is classified as a subtype of mild neurocognitive disorder (7), it is characterized by a subjective experience of cognitive decline, accompanied by objective evidence of deterioration in one or more cognitive domains compared to previous levels. However, individuals with MCI retain independence in instrumental activities of daily living and do not meet the diagnostic criteria for major psychiatric or neurological disorders (8, 9). Studies have shown that individuals with MCI are at a significantly increased risk of progressing to dementia, with approximately 20% of adults aged 65 years and older expected to develop dementia in the future (10). Therefore, as the most populous country with one of the fastest-aging populations, the early identification and effective intervention of MCI and its associated factors have become critical challenges for aging societies worldwide, particularly in China.

The influence of metabolic factors on both cognitive function and arthritis has been well-documented in numerous studies, with particular attention given to the metabolic heterogeneity of obesity (11, 12). Obesity is not a uniform metabolic condition; instead, distinct obesity phenotypes exhibit markedly different metabolic risks. For instance, individuals with metabolically healthy obesity (MHO) have excess body fat but maintain normal insulin sensitivity and favorable lipid profiles (13). In contrast, individuals with metabolically unhealthy obesity (MUO) exhibit multiple metabolic disturbances, including insulin resistance, hypertension, hyperglycemia, and dyslipidemia. While differences between these two obesity subtypes have been extensively studied in relation to certain diseases, the specific association between arthritis and MCI across different metabolic obesity phenotypes remains unclear.

In addition, mental health issues like depression may worsen cognitive decline via neuroendocrine dysregulation and chronic inflammatory processes. Depression may also mediate the link between metabolic dysfunction, arthritis, and mild cognitive impairment (MCI). However, evidence remains limited on the link between arthritis and MCI in metabolically diverse obese people, and the function of depression as a mediator in this setting is unclear. Hence, this research investigates the link between metabolic obesity phenotypes and arthritis and moderate cognitive impairment using baseline data from the 2011 CHARLS cohort. This study aims to provide scientific evidence for targeted therapies targeting obesity, arthritis, and cognitive impairment by elucidating the possible mediating role of depression in this connection.

## 2 Methods

### 2.1 Study design and participants

With the use of CHARLS data, this research used a cross-sectional design. The CHARLS research used a multistage probability sampling technique to recruit volunteers who were representative of the country. At the baseline, 17,705 people from 450 urban and rural communities in 28 Chinese provinces were polled (14). The Institutional Review Board of Peking University gave ethical clearance for the CHARLS investigation [IRB00001052-11015].

**Figure 1:**
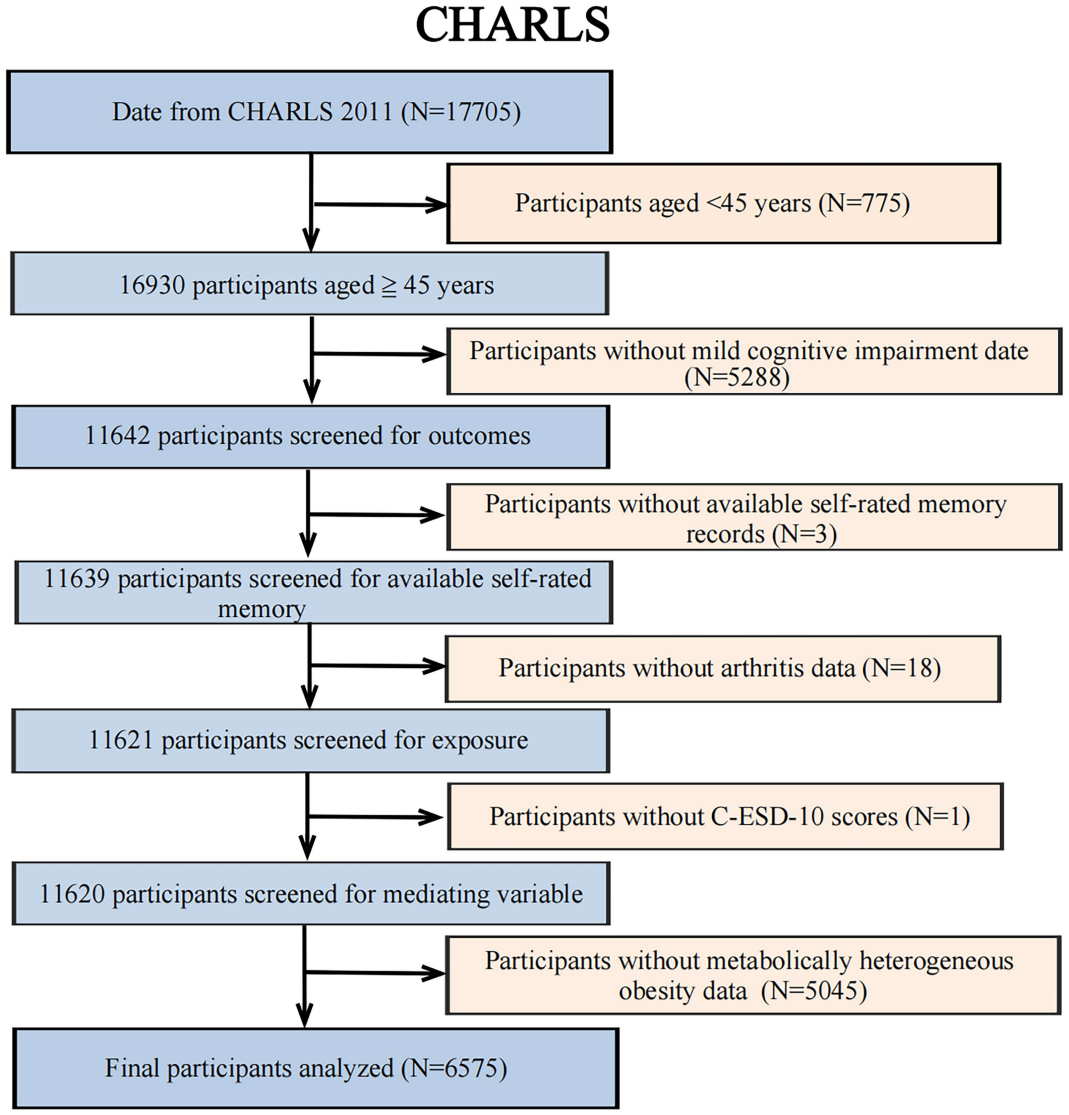
Flowchart of participant selection.

This study screened and analyzed the 2011 cross-sectional dataset from CHARLS. To ensure the robustness and credibility of the findings, a set of predefined exclusion criteria was implemented as follows: (1) respondents <45 years of age (*N* = 775); (2) respondents without MCI data (*N* = 5,288); (3) respondents without available self-rated memory records (*N* = 3); (4) respondents without available arthritis records (*N* = 18); (5) respondents without CESD-10 scores (*N* = 1); (6) respondents without MHO data (*N* = 5,045). The current study contained a final sample of 6,575 people (Figure 1).

### 2.2 Arthritis assessment

In the CHARLS 2011 questionnaire, respondents were considered to have arthritis if they had ever been diagnosed by a physician (15).

### 2.3 MCI assessment

The Word Recall Test (WRT) and the Mental Status Test (MST) are the primary measures of cognitive function included in the CHARLS 2011 questionnaire (16). The maximum score for both immediate recall and delayed recall on the WRT was 20. Eleven points were awarded for the MST, which comprised the Telephone Interview for Cognitive Status (TICS-10) and a figure-drawing task. The total score for cognitive function was obtained by summing the results of the MST and the WRT. When a person’s combined WRT and MST scores were at least one standard deviation below the age group average, the AACD criteria classified them as having mild cognitive impairment (MCI) (17, 18). The cognitive rating criteria referred to previous studies (19, 20). To further improve the accuracy of MCI diagnosis, the item of subjective memory complaint was added. The subjective memory complaints of the participants were collected through questionnaires (those who answered “average” or “poor” were considered to have subjective memory complaints), and this was used as an auxiliary diagnostic criterion.

### 2.4 MHO assessment

In the CHARLS 2011 questionnaire, participants were categorized into three groups as normal weight (18.5 ≤ BMI < 24.0 kg/m2), overweight (24.0 ≤ BMI < 28.0 kg/m2), and obesity (BMI ≥ 28.0 kg/m2) (21). Metabolic status was assessed based on four metabolic components. Participants who met two or more of the following four criteria were classified as metabolically unhealthy (22, 23): (1) elevated blood pressure, systolic blood pressure (SBP) ≥ 130 mmHg or diastolic blood pressure (DBP) ≥ 85 mmHg or use of antihypertensive drugs; (2) impaired glycemic control, fasting blood glucose (FBG) ≥ 5.6 mmol/L or glycated hemoglobin A1C (HbA1c) ≥ 6.0% or use of antidiabetic drugs; (3) elevated triglyceride (TG), TG ≥ 1.7 mmol/L or use of lipid-lowering drugs; (4) reduced high-density lipoprotein cholesterol (HDL-C), HDL-C < 1.03 mmol/L for men or <1.29 mmol/L for women or use of lipid-lowering drugs. Combined with obesity and metabolic status, participants were divided into four BMI-metabolic phenotypes as MHNW, MUNW, MHOO, and MUOO to evaluate the metabolic heterogeneity of obesity. MHNW: metabolically healthy normal weight, MUNW: metabolically unhealthy normal weight, MHOO: metabolically healthy overweight/obesity, MUOO: metabolically unhealthy overweight/obesity.

### 2.5 Mediating variable

This study investigated depression as a mediating variable CES-D-10 was utilized in the CHARLS questionnaire to assess depressive symptom severity among participants (24). A detailed overview of the CES-D-10 scoring system is provided in Supplementary Figure S1 (25). Depressive status was defined as a CES-D-10 score ≥ 12, a validated cutoff with satisfactory sensitivity and specificity for clinically significant symptoms (24).

### 2.6 Sensitivity analysis

A sensitivity analysis was carried out to ascertain the mediation model’s robustness. The initial variable that measured depression, which was either present or absent, was replaced with a continuous variable, the C-ESD-10 scores. The same set of factors was then used to conduct the mediation study.

### 2.7 Covariates

A thorough evaluation of the current literature was used to establish the variables in this investigation (26, 27). Regularized surveys were used to gather sociodemographic information, such as sex, age, residence, and degree of education. In addition, self-report questionnaires were used to gather lifestyle-related characteristics, including individuals’ histories of smoking and alcohol intake. In the covariates related to diseases, diabetes and the presence or absence of cancer (28, 29) were included. In addition, to further assess the potential impact of unmeasured confounding factors on the study results, E-value analysis was introduced. E-value analysis is a powerful tool for evaluating the extent of unmeasured confounding factors that would need to be present for the conclusions of the study results to change (30).

### 2.8 Analytical methods

R (version 4.3.2) and EmpowerStats (version 4.2) were used to conduct statistical analyses in this research. Mean ± standard deviation (SD) was used to represent continuous data, and frequencies and percentages were used to represent categorical variables. Utilization of t-tests for continuous variables and chi-square tests for categorical data to compare groups. Then, to assess the correlation between arthritis pain and MCI, multivariate logistic regression models were used. Multivariate logistic regression was used to assess the correlation between arthritis and MCI in various MHO populations, while controlling for factors such as gender, age, residence, smoking, alcohol use, and educational attainment. We computed odds ratios (ORs) and 95% CIs for every grouping. To determine if depression mediated the relationship between arthritis and MCI, we used the “mediation” package in R 4.3.2 to do a mediation study. The mediation relationship was analyzed using the Bootstrap method (31). When all three of these criteria were satisfied—(1) a statistically significant indirect impact; (2) a statistically significant overall effect; and (3) a positive percentage of the effect attributed to the mediator—then it was determined that a mediating effect was present.

## 3 Results

### 3.1 Participants characteristics

This study included 6,575 middle-aged and older individuals aged ≥ 45 years. Table 1 presents the distribution and comparison of sociodemographic characteristics, lifestyle factors, obesity heterogeneity categories, depressive symptoms, and arthritis between individuals with and without MCI. The average age of the study population was 58.18 years, with males accounting for 50.75% of the sample. A total of 2, 207 participants reported a diagnosis of arthritis.

**Table 1:** Characteristics of the study population with MCI.

Characteristics	Total sample	Mild cognitive impairment		*p* value
CHARLS database	(*N* = 6,575)	No (*N* = 1,133)	Yes (*N* = 5,442)	
Age, years	58.18 ± 8.74	57.79 ± 8.42	60.06 ± 9.96	0.004
Depressive symptoms, *n* (%)				<0.001
No	4,924 (74.89%)	1,029 (90.82%)	3,895 (71.57%)	
Yes	1,651 (25.11%)	104 (9.18%)	1,547 (28.43%)	
Gender, *n* (%)				<0.001
Male	3,337 (50.75%)	649 (57.28%)	2,688 (49.39%)	
Female	3,238 (49.25%)	484 (42.72%)	2,754 (50.61%)	
Residency, *n* (%)				<0.001
Urban	2,581 (39.25%)	536 (47.31%)	2045 (37.58%)	
Rural	3,994 (60.75%)	597 (52.69%)	3,397 (62.42%)	
Education level, *n* (%)				<0.001
Non-formal education	2,602 (39.57%)	344 (30.36%)	2,258 (41.49%)	
High school or below	3,717 (56.53%)	711 (62.75%)	3,006 (55.24%)	
Above high school	256 (3.89%)	78 (6.88%)	178 (3.27%)	
Smoking, *n* (%)				0.031
No	3,839 (58.39%)	629 (55.52%)	3,210 (58.99%)	
Yes	2,736 (41.61%)	504 (44.48%)	2,232 (41.01%)	
Drinking, *n* (%)				0.026
No	3,845 (58.48%)	629 (55.52%)	3,216 (59.10%)	
Yes	2,730 (41.52%)	504 (44.48%)	2,226 (40.90%)	
Arthritis, *n* (%)				<0.001
No	4,368 (66.43%)	884 (78.02%)	3,484 (64.02%)	
Yes	2,207 (33.57%)	249 (21.98%)	1958 (35.98%)	
MHO, *n* (%)				0.265
MHNW	1993 (30.31%)	339 (29.92%)	1,654 (30.39%)	
MHOO	867 (13.19%)	155 (13.68%)	712 (13.08%)	
MUNW	1,609 (24.47%)	255 (22.51%)	1,354 (24.88%)	
MUOO	2,106 (32.03%)	384 (33.89%)	1722 (31.64%)	
Diabetes, *n* (%)				0.891
No	6,122 (93.11%)	1,056 (93.20%)	5,066 (93.09%)	
Yes	453 (6.89%)	77 (6.80%)	376 (6.91%)	
Cancer, *n* (%)				0.207
No	6,520 (99.16%)	1,120 (98.85%)	5,400 (99.23%)	
Yes	55 (0.84%)	13 (1.15%)	42 (0.77%)	

MCI, Mild cognitive impairment; MHO, Metabolically heterogeneous obesity; MHNW, metabolically healthy normal weight; MUNW, metabolically unhealthy normal weight; MHOO, metabolically healthy overweight/obesity; MUOO, metabolically unhealthy overweight/obesity.

Notably, age, depressive symptoms, residency, education level, arthritis, were significantly associated with MCI.

### 3.2 Associations of arthritis with MCI

According to Table 2, all three models demonstrated a significant positive relationship between arthritis and MCI, and this relationship was statistically significant. In Model 1, the OR was 2.00 (95% CI = 1.72–2.32, *p* < 0.001). In Model 2, the OR was 1.92 (95% CI = 1.65–2.23, *p* < 0.001). And after adjusting for all confounding factors, Model 3 showed an OR of 1.86 (95% CI = 1.60–2.17, *p* < 0.001), indicating a positive correlation.

**Table 2:** Associations of arthritis with MCI.

CHARLS database	OR (95% CI)		
Arthritis	Model 1	Model 2	Model 3
No	Reference	Reference	Reference
Yes	2.00 (1.72, 2.32)	1.92 (1.65, 2.23)	1.86 (1.60, 2.17)
*p* value	<0.001	<0.001	<0.001

Model 1 adjust for: none. Model 2 adjust for: gender, age, residency. Model 3 adjust for: gender, age, residency, smoking, drinking, education level, diabetes, cancer.

### 3.3 Stratified analysis by BMI–metabolic phenotypes

The association between arthritis and MCI across different MHO populations is shown in Table 3. Interestingly, all subgroups showed a positive correlation, but only the MUOO group (OR = 1.51, 95% CI = 1.18–1.92) demonstrated a statistically significant and the strongest positive correlation. No significant differences were observed in the MHNW group (OR = 1.08, 95% CI = 0.85–1.38), MHOO group (OR = 1.23, 95% CI = 0.81–1.87), or MUNW group (OR = 1.16, 95% CI = 0.90–1.49).

**Table 3:** Stratified analysis of associations between arthritis and MCI in MHO people.

MHO	CHARLS database *N* (%)	OR (95% CI)	*p* value
111MHNW	1993	1.63 (1.24, 2.15)	0.0005
111MHOO	867	1.82 (1.18, 2.81)	0.0063
111MUNW	1,609	1.68 (1.23, 2.31)	0.0013
111MUOO	2,106	2.27 (1.73, 2.97)	<0.0001

MHNW, metabolically healthy normal weight; MUNW, metabolically unhealthy normal weight; MHOO, metabolically healthy overweight/obesity; MUOO, metabolically unhealthy overweight/obesity.

### 3.4 Mediating role of depressive symptoms in metabolically unhealthy overweight/obesity people

**Figure 2:**
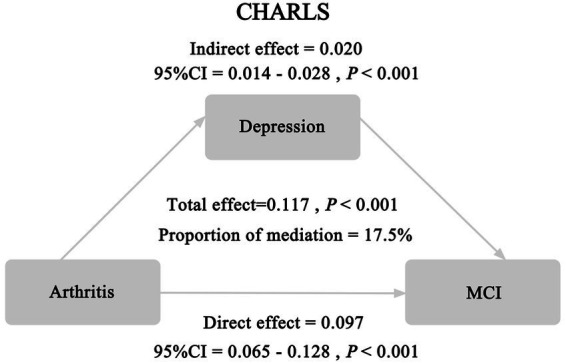
Mediating role of depressive symptoms in metabolically unhealthy overweight/obesity people.

Mediating effect analysis revealed that depression partially mediated the association between arthritis and MCI in metabolically unhealthy overweight/obesity people, as shown in Figure 2. Depression accounted for 17.5% of the association between arthritis and MCI. The indirect effect, direct effect, and total effect of arthritis on MCI were all statistically significant.

### 3.5 Findings from the sensitivity analysis

**Figure 3:**
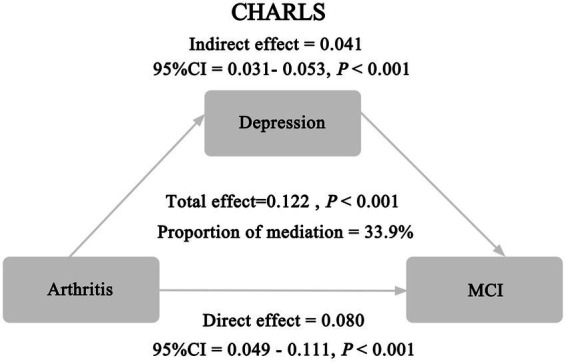
Sensitivity analysis.

Results indicated that the mediation effect remained significant when depression was treated as a continuous variable (Figure 3). Depression accounted for 33.9% of the association between arthritis and MCI. The indirect effect (0.041, 95% CI = 0.031–0.053, *p* < 0.001), direct effect (0.080, *p* < 0.001), and total effect (0.122, *p* < 0.001).

Further E-value analysis showed that even if there were unmeasured confounding factors, their impact on the results would have to be very high to change our conclusions, with a specific E-value of 3.12. The E value reported in this study corresponds to the effect estimate.

## 4 Discussion

This study demonstrated a positive association between arthritis and MCI, which remained statistically significant even after comprehensive adjustment for covariates. Furthermore, stratified analyses based on metabolic obesity phenotypes revealed a consistent positive relationship between arthritis and MCI across all subgroups; however, this association reached statistical significance solely in the MUOO subgroup. Further analysis demonstrated that depression partially mediated the link between arthritis and mild cognitive impairment in the MUOO population, with an estimated mediation proportion of 17.5%. These findings highlight the complicated interrelationship between arthritis and MCI within metabolically heterogeneous obese populations.

Multiple studies have reported the complex relationship between inflammation and cognitive impairment (32, 33). According to a cross-sectional study in the United States, arthritis was negatively associated with cognitive improvement, and this relationship remained significant after adjusting for potential confounders (OR:0.796, 95% CI:0.649–0.975; OR:0.769, 95% CI:0.611–0.968). This finding aligns with the results of the current study. The precise links between arthritis and cognitive impairment have yet to be fully elucidated (34). Some researchers have proposed several potential links, including chronic pain, depression and anxiety, inflammatory processes, and genetic factors (35, 36). Chronic pain and reduced physical activity caused by arthritis may directly influence the secretion of brain-derived neurotrophic factor (BDNF). A study involving 334 individuals with cognitive decline reported that those with limited mobility had a 47.6% increased risk of developing MCI (37). Moreover, a widely accepted hypothesis suggests that arthritis, as a chronic inflammatory disease, may affect cognitive function through multiple links (38). Alexandre et al. suggested that some pro-inflammatory cytokines—interleukin-6 (IL-6) in particular—may traverse the blood–brain barrier and exert regulatory effects on neuronal activity (39).

The association between arthritis and MCI was found to be partially explained by the mediating effect of depression. This may be explained by the inflammation–depression–cognition axis: chronic pain and functional impairment in patients with arthritis can predispose them to depression, such as feelings of worthlessness or hopelessness and reduced physical activity. Depression, in turn, may upregulate pro-inflammatory cytokines (e.g., TNF-*α*), forming a positive feedback loop that adversely affects cognitive function (35). To our knowledge, few studies have examined the association between arthritis and MCI specifically in the metabolically unhealthy overweight/obese population, and our findings suggest that depression may mediate this relationship. The findings suggest that high inflammatory activity in patients with arthritis—such as elevated Disease Activity Score-28 (DAS28) and C-reactive protein (CRP) levels—is significantly associated with impairments in visuospatial ability, memory, and executive function (40), Individuals with metabolically unhealthy overweight/obesity (MUOO) are characterized by a state of low-grade systemic inflammation, which, when compounded by the inflammatory burden of arthritis, may be associated with neurodegenerative processes and a higher likelihood of MCI (41).

The main strength of this study lies in its use of an observational cross-sectional design to explore the associations and mediation analyses among variables within the large and nationally representative CHARLS dataset. This design was chosen because it allows for the simultaneous examination of the relationships between arthritis, MCI, and potential modulators such as depression. This supports the applicability of our findings and may inform the design of future longitudinal studies, thereby laying the groundwork for further research. Given the observed association between arthritis and MCI among individuals with metabolically unhealthy overweight/obesity, screening for depression and MHO status may hold significant clinical and public health relevance for individuals affected by arthritis in both clinical and community settings. Healthcare providers and practitioners should remain highly vigilant for potential MCI in the aging population with arthritis and metabolically unhealthy overweight/obesity, particularly when depressive symptoms are present.

This research has certain limitations. The data originated from a Chinese population sample, perhaps limiting the generalizability of the results. Future research using samples from more nations is necessary to confirm the validity of these results. Certain characteristics included in this research were obtained from self-reports. Supplied by the participants, potentially resulting in recollection bias. Despite the involvement of expert clinical professionals, many middle-aged and older adults with inadequate knowledge and health literacy may still struggle to appropriately comprehend their own health situation. In this study, the diagnosis of arthritis primarily relied on the self-reports of the participants.

This study employed a cross-sectional design, which to some extent limits our inference regarding causality and the temporal sequence of events. The exclusion of samples due to missing data in this study may introduce selection bias. To further validate these hypotheses, future research needs to adopt a longitudinal design, tracking changes in individuals over a longer period of time to clarify causality and the temporal order of events.

## 5 Conclusion

Our findings reveal a positive link between arthritis and mild cognitive impairment among individuals with metabolically unhealthy overweight/obesity, and underscore the significant mediating effect of depression in this relationship. For adults in midlife and later life with arthritis and metabolically unhealthy overweight/obesity, we recommend that clinicians and community health workers adopt an integrated strategy combining metabolic regulation (e.g., improving insulin sensitivity), anti-inflammatory treatment (e.g., TNF inhibitors that may delay the progression of MCI), and psychological intervention (e.g., depression screening), to break the vicious cycle of inflammation, depression, and cognitive decline.

## Data Availability

Publicly available datasets were analyzed in this study. This data can be found here: http://charls.pku.edu.cn/en.

## Supplementary material

SUPPLEMENTARY FIGURE S1 Depression assessment.
