# Supplementary figures and images for: The relationship between arthritis and mild cognitive impairment in different obese metabolic heterogeneity populations-the mediating role of depression
Source: Front Nutr. 2026 Feb 2;13:1653086. doi: 10.3389/fnut.2026.1653086 (PMC12908586; doi:10.3389/fnut.2026.1653086)

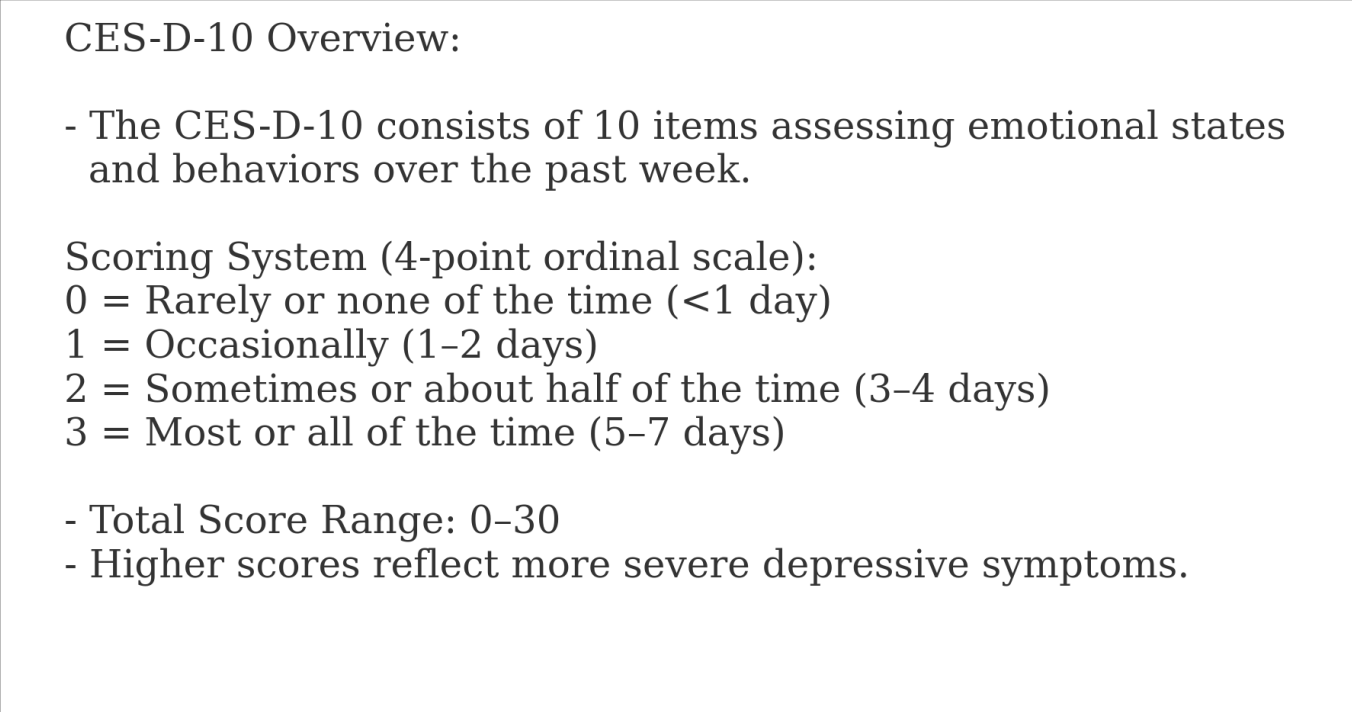

Supplement: SUPPLEMENTARY FIGURE S1 — Depression assessment. [file Image_1.tiff]
